# Smart Sensing Technology for Human Activity Recognition

**DOI:** 10.3390/s26175363

**Published:** 2026-08-25

**Authors:** Giorgio Biagetti, Paolo Crippa

**Affiliations:** DII—Dipartimento di Ingegneria dell’Informazione, Università Politecnica delle Marche, Via Brecce Bianche 12, I-60131 Ancona, Italy; p.crippa@univpm.it

## 1. Introduction

Human activity recognition (HAR) aims to recognize specific user activities or discover long-term patterns from a set of observations in real-life contexts, captured through a variety of sensors that can span many domains (inertial, bioelectrical, optical, etc.). Its potential of being non-invasive in everyday life makes it one of the most promising enabling technologies for ambient assisted living [1].

Sensing technology for HAR involves a plethora of research topics, including both hardware and software aspects for the implemented systems. On the hardware side, advances in micro- and nanoelectronics, semiconductor devices and materials, as well as communication front-ends, have enabled the development of lightweight, low-power wearable sensors for HAR in an increasingly user-friendly manner [2,3,4,5,6,7,8]. From a software perspective, due to their ability to model sequential data and capture temporal dependencies in time-series data, deep learning (DL) architectures based on recurrent neural networks (RNNs) have become a useful tool for HAR [9,10,11,12,13,14,15,16,17].

The aim of this Special Issue is to collect relevant papers that deal with innovative aspects of HAR systems, including sensor hardware design, all stages of data processing (from acquisition to the presentation of the results thus acquired), with a focus on intelligent algorithms that are able to automate the classification or recognition of activities, or improve the quality of the acquired information. We thus present papers that describe innovative developments in the acquisition of signals related to a person’s activity, and the interpretation of the data through automated techniques such as machine learning and artificial intelligence [18,19,20,21,22,23,24,25].

## 2. Overview of Published Papers

In the first contribution, the authors adopt an open-set recognition framework for HAR, wherein not all activity classes need to be defined during the training phase. This framework enables the extension of existing HAR classifiers (based on head-mounted devices) to detect previously unseen activities by incorporating lightweight, post hoc open-set mechanisms, without altering the model architecture or the training procedure. Furthermore, an extensive empirical evaluation is presented, based on a “leave-one-activity-out” validation protocol applied to two datasets containing IMU signals captured via smart glasses: a proprietary dataset and the public UCA-EHAR dataset. A lightweight one-dimensional convolutional neural network was trained to classify six-axis IMU data corresponding to common activities. HAR performance was assessed in an open-set setting using various low-computational-cost methods operating in the logit space, including maximum logit, Gaussian mixture models, kernel density estimation, OpenMax, and nearest neighbor distance ratio. Robust recognition of unknown activities was achieved, yielding an area under the ROC curve exceeding 0.8. These results highlight the potential of low-complexity open-set approaches for real-time HAR on resource-constrained wearable platforms, fostering the development of sensor-based recognition systems that are both adaptive and reliable for deployment in real-world scenarios.

The second contribution proposes a lightweight, non-invasive system capable of identifying water flow sources to enable human activity recognition (HAR), specifically for activities of daily living in smart home environments, without requiring modifications to the plumbing system. The proposed system is particularly significant for facilitating the early detection of cognitive decline by analyzing variations in bathroom usage patterns. The system employs a single microphone to capture ambient sounds within a room and identifies active water sources based on their acoustic signatures. Audio signals are converted into time-resolved spectrograms, processed via convolution in both the time and frequency domains, and subsequently classified using a neural network. This approach allows for both the identification of specific water sources (even when used simultaneously) and the measurement of usage duration, achieving an overall accuracy of 90.6%. The study examines four common bathroom fixtures: the toilet, bidet, shower, and washbasin. The proposed system is adaptable to various environments and does not require changes to plumbing infrastructure, making it suitable for smart home and digital health applications.

The third contribution presents a dynamic hand gesture classification system based on long short-term memory (LSTM) neural networks deployed on a resource-constrained STM32L4 microcontroller. Using a public dataset comprising 20 hand gestures performed by 10 subjects and recorded through a wrist-worn three-axis accelerometer, the proposed solution achieved an accuracy of 90.25%. The inference time was 418 ms for 4 s input sequences, corresponding to an average CPU utilization of approximately 10%. The results demonstrate that the proposed architecture satisfies real-time processing requirements while maintaining competitive recognition performance. Furthermore, the network occupies only about 25% of the memory available for model parameters, highlighting its suitability for low-power and wearable smart sensing applications.

The fourth contribution investigates HAR using WiFi channel state information (CSI) signals through a lightweight architecture based on a single-layer gated recurrent unit (GRU) enhanced with an attention mechanism. More in detail, the proposed approach employs a GRU-based architecture for time-series feature extraction, followed by a self-attention module that emphasizes the most informative activity-related features. Subsequently, pruning is applied to remove redundant weights, substantially improving efficiency with minimal impact on performance. An ablation study further confirms that the proposed pruning strategy preserves accuracy despite a significant reduction in model complexity. To address the limited size of available WiFi CSI datasets, several data augmentation techniques are incorporated. Combined with pruning, these methods mitigate overfitting, improve generalization, and further reduce computational requirements. Additional experiments evaluate the contribution of different augmentation strategies, demonstrating their effectiveness in maintaining high performance under resource-constrained conditions. Compared with the state-of-the-art, this approach reduces the model size from 252.10 M to 0.0578 M parameters and lowers computational cost from 18.06 GFLOPs to 0.01 GFLOPs, making it particularly suitable for practical HAR applications.

The fifth contribution investigates the use of an ear-worn motion sensor for HAR in healthcare applications. Data were collected from 50 healthy participants performing daily activities, including lying, sitting/standing, walking, climbing stairs, and running. Both traditional machine learning (ML) methods and advanced deep learning (DL) models were assessed for activity classification. The results show that the DL approaches achieve up to 98% accuracy, while remaining robust to sensor placement and orientation variations, eliminating the need for calibration. These findings confirm the ear as a reliable location for activity monitoring. By integrating motion sensing with in-ear vital sign monitoring, the proposed approach enables comprehensive health assessment and supports applications in telemonitoring, personalized healthcare, and sports performance analysis.

The sixth contribution presents Wi-CHAR, a WiFi-based cross-domain HAR system designed to operate effectively across different environments with limited training data. The proposed approach achieves high recognition accuracy and strong generalization capabilities over large areas while requiring only a small number of samples. By addressing performance degradation commonly observed in cross-domain scenarios, Wi-CHAR eliminates the need for model retraining when data collection in real-world environments is constrained. The system first employs a dynamic WiFi sensor selection strategy to improve sensing reliability and data quality in multi-device deployments. It then applies the MF-DBSCAN clustering algorithm to identify and correct anomalous data, enhancing the robustness of the activity recognition process. In addition, the so-called Re-PN module dynamically updates feature prototype weights, enabling effective adaptation to new users and environments while distinguishing reliable samples from noisy ones. Experimental evaluations conducted in real-world scenarios demonstrate the effectiveness of the proposed framework. Despite limited labeled training data, Wi-CHAR achieves an average cross-domain recognition accuracy of 93.2% in multi-WiFi link environments. These results highlight its potential for applications in smart homes, healthcare monitoring, and elderly care.

The seventh contribution presents an IoT-based exercise promotion system designed to encourage healthier lifestyles among people living alone. Built on an agent-oriented IoT architecture, the system uses autonomous IoT agents to monitor user activity, estimate contextual conditions, and provide personalized exercise recommendations based on user behavior, environment, and preferences. User feedback is leveraged to continuously improve recommendation quality. The system was evaluated through real-world experiments conducted in participants’ homes. Experimental results show that the system accurately recognizes user conditions, adapts to preferences within one to two weeks, and increases motivation to exercise. Its decentralized design enhances flexibility and scalability while enabling low-cost deployment through off-the-shelf IoT devices and smartphones.

The eighth contribution investigates the use of a single 2D light detection and ranging (2D-LIDAR) sensor positioned at hip height for privacy-preserving person identification and activity recognition in indoor environments, with the goal of supporting socially assistive robots for older adults. Human contours are extracted from 2D-LIDAR point clouds and analyzed using DL models for resident identification and activity estimation. Two DL approaches based on LSTM and image classification (VGG16) were evaluated on data collected from four participants performing activities such as walking, sitting, standing, and opening/closing a door. The VGG16-based model achieved the best performance, reaching 89.7% accuracy in person identification and 97.9% accuracy in activity recognition. The results demonstrate that a single 2D-LIDAR sensor can effectively identify residents and recognize common indoor activities while preserving privacy, making it a promising solution for smart home monitoring and socially assistive robotics.

The ninth contribution presents SkeletonCLIP++, an enhanced framework for HAR that exploits semantic information beyond conventional label-based approaches. The model introduces three key components: ‘weighted frame integration’, which generates richer video representations by assigning different importance to individual frames; ‘contrastive sample identification’, which improves discrimination between visually similar actions by leveraging challenging negative samples; and ‘BERT text encoder integration’, which strengthens semantic understanding through the use of a pre-trained BERT encoder. Experiments on three widely used datasets demonstrate consistent performance improvements, particularly on smaller datasets. The results show that the combination of semantic-aware video representations, enhanced contrastive learning, and advanced text encoding enables more accurate recognition of complex human actions and better differentiation between closely related activities. Overall, SkeletonCLIP++ provides a more effective and semantically informed approach to HAR by improving the alignment between visual and textual representations.

The tenth contribution investigates the feasibility of remotely assessing the quality of upper-limb rehabilitation exercises performed at home by stroke survivors. Using a wrist-worn inertial measurement unit (IMU), participants performed functional tasks both in a laboratory setting and independently at home. Laboratory recordings were used as reference data, while movement quality was evaluated through a dynamic time warping (DTW) algorithm and therapist-provided labels. The results showed that the proposed approach can accurately distinguish between correct and compensatory movement patterns, achieving a classification accuracy of 92% and an F1-score of 0.95 across all tasks. Overall, this study supports the development of a remote movement-quality monitoring system that can help improve home rehabilitation, provide objective feedback, and enhance the effectiveness of post-stroke recovery programs.

The eleventh contribution investigates the feasibility of human posture recognition through passive RF sensing using frequency-modulated (FM) radio signals received at multiple locations. To this end, the authors developed a testbed based on software-defined radios that operates in FM radio bands and supports both offline analysis and real-time posture classification. The proposed framework includes feature extraction and selection, followed by the application of conventional ML classifiers. Experimental results show that simple classification methods can effectively distinguish different postures, achieving approximately 90% accuracy. Among the evaluated ML approaches, a support vector machine (SVM) with a radial basis function (RBF) kernel delivered the best performance, reaching 87.9% accuracy, 93.9% specificity, and an F1-score of 87.9%. Overall, the study demonstrates that passive sensing of FM radio signals can provide reliable human posture recognition without requiring users to wear devices, highlighting its potential for future indoor monitoring and HAR applications.

The twelfth contribution presents a DL framework for grasped object recognition in collaborative robotics, enabling robots to infer human intentions from hand configurations during shared tasks. The approach combines MediaPipe Hands for extracting hand and finger keypoints from RGB images with a multi-class classifier that predicts the object being manipulated. Two DL architectures, a convolutional neural network (CNN) and a transformer, were evaluated on a newly collected dataset involving multiple users and recording sessions. Performance was assessed using accuracy, precision, recall, and F1-score, with particular attention to the models’ ability to generalize across users and sessions. The results demonstrate that hand keypoints provide effective information for object recognition, highlighting the potential of the proposed approach for intention prediction in human–robot collaboration. At the same time, the study identifies challenges related to cross-user and cross-session generalization, emphasizing the need for continuous model adaptation, retraining, and personalized strategies to improve robustness in real-world applications.

The thirteenth contribution proposes a distributed mmWave MIMO radar framework for orientation-independent HAR and fall detection. Unlike conventional systems relying on a single monostatic radar, which struggle to capture motion occurring orthogonally to the radar’s line of sight, the proposed approach employs two monostatic radars positioned at right angles to provide complementary views of human movements. Micro-Doppler signatures acquired from both radars are processed to compute mean Doppler shifts and extract statistical, temporal, and frequency-domain features. These features are then combined through feature-level fusion and classified using an SVM. The approach was evaluated on a dataset containing more than 1350 activity trials performed by six participants across multiple orientations. The experimental results show an overall classification accuracy between 98.31% and 98.54%, outperforming traditional single-radar systems by approximately 6%. Overall, the proposed system demonstrates that combining complementary radar perspectives significantly improves the robustness and accuracy of RF-based HAR, enabling reliable recognition of human activities and falls regardless of subject orientation.

Finally, the fourteenth contribution is a perspective paper that examines the development of HAR systems for smart homes, emphasizing the need for tailored solutions that support automated assistance, health monitoring, and long-term behavioral analysis. Given the highly personalized nature of home environments, the authors argue that effective HAR systems cannot rely on generic off-the-shelf solutions and must instead evolve throughout their operational lifecycle. To address this challenge, the paper introduces a lifespan framework that defines the key stages involved in designing, deploying, and continuously improving HAR systems. Each stage is linked to realistic smart-home application scenarios and supported by data-driven methodologies that enable the system to adapt over time with minimal supervision. The proposed framework also identifies the main technical challenges and outlines the components required to build robust and scalable HAR solutions. By providing a structured view of the HAR lifecycle, the work offers practical guidance for the development of next-generation smart-home activity recognition systems capable of supporting resident well-being and independent living.

## 3. Conclusions

This Special Issue presents a collection of fourteen articles addressing various aspects of smart sensing technologies used for human activity and gesture recognition, offering an overview of the state of the art and future prospects in this field. We hope that the contributions gathered here will provide valuable insights for the design and implementation of wearable smart sensors and HAR systems, serving as a useful reference for future work in this rapidly evolving field of research and application.

We wish to express our sincere gratitude to the authors who shared their expertise and knowledge in this Special Issue, and also to the anonymous reviewers who dedicated their time and professionalism to providing high-quality suggestions and feedback, thereby making the realization of this strong collection of papers possible.
